# Complete Genome Sequence of *Staphylococcus aureus* Bacteriophage pSa-3

**DOI:** 10.1128/genomeA.00182-17

**Published:** 2017-05-25

**Authors:** Sang Guen Kim, Jin Woo Jun, Sib Sankar Giri, Saekil Yun, Hyoun Joong Kim, Cheng Chi, Sang Wha Kim, Se Chang Park

**Affiliations:** Laboratory of Aquatic Biomedicine, College of Veterinary Medicine and Research Institute for Veterinary Science, Seoul National University, Seoul, Republic of Korea

## Abstract

*Staphylococcus aureus* phage pSa-3, isolated in South Korea from a sewage sample, has a 137-kb genome with 29% G+C content. This phage was targeted to control the bacteria in clinical isolates (mainly from skin lesions) and can be used in the decolonization of *S. aureus*.

## GENOME ANNOUNCEMENT

The colonization of *Staphylococcus aureus* on the skin of patients with atopic dermatitis is universal (1) and is considered a factor that aggravates lesions (2). So far, a number of phages that infect *S. aureus* have been isolated to cope with the multidrug-resistant strains of the species (3).

The morphology of pSa-3, a bacteriophage isolated from sewage water, was analyzed using transmission electron microscopy. pSa-3 belongs to the *Myoviridae* family; it has a 74-nm-diameter head and a contractile tail that is 106 nm long.

The phage was propagated by the conventional top agar method and purified using polyethylene glycol precipitation. DNA was extracted using a phenol extraction method (4) and sequenced using the Illumina HiSeq 2500 platform at Genotech (Daejeon, South Korea). A total of 27,545,920 reads (2,782,137,920 bp) were trimmed and assembled using CLC Genomics Workbench version 6.5.1. The average coverage of sequence was 1,387×. Open reading frame (ORF) prediction and annotation were conducted using Glimmer version 3.02 (5), Prodigal version 1.20 (6), GeneMarkS version 4.08 (7), and protein BLAST (8), respectively, and confirmed using the Rapid Annotations using Subsystems Technology (RAST) server (9). tRNAs were predicted using tRNAscan-SE version 2.0 (10), and the nucleotide homology of pSa-3 was determined using EMBOSS Stretcher (11).

The genome of pSa-3 comprised linear double-stranded DNA that was 137,836 bp long, with 29% G+C content. In a comparison of this genome to those of various Twort-like phages (A5W, Staph1N, P4W, 676Z, A3R, MSA6, and G1), the nucleotide homology was approximately 90 to 93%. Of the 208 ORFs predicted, 102 encoded hypothetical proteins. The remaining 106 ORFs were classified into 5 groups: DNA metabolism (Rep protein, exonuclease, DNA repair protein, DNA polymerase A, nucleoside triphosphate pyrophosphohydrolase, nucleoside 2-deoxyribosyltransferase, DNA transfer protein, DNA primase, DNA ligase, and DNA helicase), phage structure (tail protein, major tail protein, tail morphogenetic protein, tail assembly chaperone, tail tube protein, tail sheath protein, structural protein, prohead protease, membrane protein, baseplate protein, major capsid protein, capsid protein, and scaffold protein), packaging (terminase large subunit, portal protein, and HNH endonuclease), lytic properties (*N*-acetylmuramoyl-l-alanine amidase, holin, and tail lysin), and additional functions (AAA family ATPase, putative immunoglobulin-like protein, BofL, glycerophosphoryl diester phosphodiesterase, Iro, LysM domain-containing protein, MbpB, UboA, and integration host factor).

### Accession number(s).

The genome sequence of pSa-3 was deposited in GenBank under the accession number KY581279.
